# Supplementary material for: Predicting gene regulatory networks from cell atlases
Source: Life Sci Alliance. 2020 Sep 21;3(11):e202000658. doi: 10.26508/lsa.202000658 (PMC7536823; doi:10.26508/lsa.202000658)
Supplement: Supplementary file 1 [file LSA-2020-00658_Supplemental_Data_1.docx]

***Predicting gene regulatory networks from cell atlases***

Andreas Fønss Møller^1^ and Kedar Nath Natarajan^1,2, #^

^1^ Functional Genomics and Metabolism Unit, Department of Biochemistry and Molecular Biology, University of Southern Denmark, Denmark

^2^ Danish Institute of Advanced Study (D-IAS), University of Southern Denmark, Denmark

^#^ Correspondence: [knn@bmb.sdu.dk](mailto:knn@bmb.sdu.dk)

**Supplementary Methods**

**Datasets**

Tabula Muris: The Tabula Muris scRNA-seq dataset contains single-cells profiled using both 3’end 10x Chromium and full-length Smart-Seq2 [[1]](https://paperpile.com/c/JqQ5C9/7gzKn). The data was retrieved through the data portal (<https://figshare.com/projects/Tabula_Muris_Transcriptomic_characterization_of_20_organs_and_tissues_from_Mus_musculus_at_single_cell_resolution/27733>). The data contained we obtained 1,23,878 single-cells. The Smart-seq2 dataset consists of 53,760 single-cells from 18 tissues classified into 81 cell types, while the 10x Chromium contains 70,118 single-cells from 12 tissues classified into 55 cell types. After filtering the non-annotated cell types, we obtained 44,779 and 54,865 single-cells from Smart-seq2 and 10x Chromium respectively. The annotated cell types from Smart-seq2 and 10x Chromium are referred to as author assigned cell-type labels.

Mouse Cell Atlas: The Mouse Cell Atlas (MCA) [[2]](https://paperpile.com/c/JqQ5C9/Fnvuu) scRNA-seq dataset contains single-cells profiled using authors 3’ end microwell method. The data was retrieved through the data portal (<https://figshare.com/articles/MCA_DGE_Data/5435866>). After filtering non-annotated cells types, we obtained 2,33,994 single-cells from 38 tissues classified into 760 cell types. The annotated cell types from across 3’ end microwell method are referred to as author assigned cell-type labels.

Myeloid Differentiation: The myeloid differentiation dataset contains 382 wildtype (9 cell types) and 62 Irf8^-/-^ cells [[3]](https://paperpile.com/c/JqQ5C9/kiw2W). The scRNA-seq expression matrices were retrieved from data portal (<https://www.dropbox.com/sh/yqlclftyolwqy7y/AADVD-_IOqpXQx8PlWcywMypa?dl=0>) [[4]](https://paperpile.com/c/JqQ5C9/LKUvC).

**Data Processing**

Data normalization and scaling: We use Scanpy (version 1.4) for normalization of all datasets [[5]](https://paperpile.com/c/JqQ5C9/KYRMm) using the pre-processing functions for cell library size (scanpy.pp.normalize_per_cell) and log-transformation (scanpy.pp.log1p). We regress the variance arising from variable library size and mitochondrial gene count fraction, and scale genes (zero mean and unit variance) using in-built functions (scanpy.pp.regress_out and scanpy.pp.scale respectively). The Highly Variable Genes (HVGs) for each dataset are calculated using in-built functions (scanpy.pp.highly_variable_genes) with default parameters.

Pseudobulk: For creating pseudobulk cells, we randomly sampled 50 cells from author assigned reference cell-type within a given tissue. Only genes with non-zero counts are used for averaging. This approach potentially removes author assigned cell-types consisting of fewer than 50 cells (very rare cells).

Cell cycle stage prediction: The cell cycle stage prediction is performed using Scanpy function (scanpy.tl.score_genes_cell_cycle) to score S and G2M specific genes. Each single-cell has a S- and G2M-score, and is assigned respectively based on the highest scoring class. If neither S-score nor G2M-score exceeds 0.5, the cells are assigned as G1 phase. The reference cell-cycle phase marker genes [[6]](https://paperpile.com/c/JqQ5C9/tX2PW) used for scoring can be found here (<https://github.com/theislab/scanpy_usage/blob/master/180209_cell_cycle/data/regev_lab_cell_cycle_genes.txt>)

**Mapping author assigned cell-type labels to common reference**

Reference cell types: We first devise a common reference for mapping different author assigned cell-type labels. We choose Tabula Muris 10x cell-type labels as reference cell types as it has the fewest annotated cell types for effective integration. The reciprocal reference using either Tabula Muris Smart-seq2 or Mouse Cell Atlas lead to unresolved and undefined cell types and poor mapping. We manually curated the reference cell types to 7 cell groups (Supplementary Fig 3B).

scMAP: We map both Tabula Muris Smart-seq2 and Mouse Cell Atlas to Tabula Muris 10x separately using scMAP (version 1.4.1) with default parameters [[7]](https://paperpile.com/c/JqQ5C9/2y9jE). We use the function ‘selectFeatures’ for identifying features and used the common feature set (Intersection) for mapping and therefore avoided. This further reduces the contribution of cell types either identified in single atlas or without any common features with reference cell types. For example, none of the Mouse Cell Atlas single-cells mapped to reference cell type “Keratinocytes” in Tabula Muris 10x. Similarly, none of the Tabula Muris Smart-seq2 single-cells mapped to reference cell type “Duct epithelial cells” in Tabula Muris 10x. In the last step, we further exclude non-mapping cells. The remaining single-cells from Tabula Muris 10x (54,865 cells), Smart-seq2 (38,888 cells) and Mouse Cell Atlas (150,889 cells) are used for regulon inference.

**Inferring gene regulatory networks**

Feature selection for pySCENIC: To retain a large but stringent feature size while accounting for technical atlas differences, we select the features that are expressed in 10% of pseudobulk cells for downstream analysis (Fig. 1B). Similarly, we select genes expressed in 10% wildtype cells (1002 genes) from the myeloid differentiation dataset [[3]](https://paperpile.com/c/JqQ5C9/kiw2W).

Dataset preprocessing for pySCENIC: The raw datasets are normalised using Scanpy pre-processing functions for cell library size (scanpy.pp.normalize_per_cell) and log-transformed (scanpy.pp.log1p). No additional scaling of genes was performed.

Running pySCENIC: We implement the 3 steps for pySCENIC pipeline [[8]](https://paperpile.com/c/JqQ5C9/Y5vWg). First, GRNboost is run on filtered expression matrix using list of transcription factors (<https://resources.aertslab.org/cistarget/motif2tf/motifs-v9-nr.mgi-m0.001-o0.0.tbl>). Secondly, RcisTarget is used to infer direct targets using ‘mm9-500bp-upstream-7species’ and

‘mm9-tss-centered-10kb-7species’ (<https://resources.aertslab.org/cistarget/>). The defined regulons are transcription factors (TF) and their direct target genes harbouring significant TF motif enrichment. Thirdly, regulon activity score (RAS) is calculated using AUCell as the area under the recovery curve of all genes identified within the regulon. All the steps are run with default parameters. The regulon inference identifies 233 regulons in Tabula Muris 10x (median composition of 141.5 genes), 222 regulons in Tabula Muris Smart-seq2 (median composition of 195 genes) and 222 regulons in Mouse Cell Atlas (median composition of 151 genes).

Similarly, we identify 154 regulons (median composition 93.5 genes) from wildtype cells in myeloid differentiation dataset [[3]](https://paperpile.com/c/JqQ5C9/kiw2W). We also separately ran pySCENIC pipeline on Monocytes (191 regulons, median 48 genes), Granulocytes (181 regulons, median 54.5 genes) and Irf8^-/-^ cells (136 regulons, median 69.5 genes) respectively. To specifically infer Irf8 regulon activity (Supplementary Fig 22D) in both wildtype and Irf8^-/-^ cells, we repeated AUCell 50 times and used the averaged activity score.

**Cell Type similarity based on regulon activity**

Spearman correlation: We calculated pseudobulk cell-to-cell spearman correlation coefficients based on RAS to quantify cell type similarity using ‘scipy.stats.spearmanr’ (version 1.1.0). The pseudobulk spearman correlation coefficients are classified by hierarchical clustering using ‘seaborn.clustermap’ function (version 0.9.0) with default parameters. The force directed graphs only link edges where the spearman correlation coefficients are greater than 0.5.

**Embedding**

PCA: Principal Component Analysis is performed on RAS using ‘scanpy.tl.pca’ with default parameters.

UMAP: We performed Uniform Manifold Approximation and Projection [[9]](https://paperpile.com/c/JqQ5C9/XBPrp) using the Scanpy function ‘scanpy.tl.umap’ with default parameters.

**Comparison of RAS and regulon composition between single- and pseudobulk cells**

PCA and cluster centers: To compare the RAS between single- and pseudobulk bulks, we first plotted pseudobulk cells on PCA (sklearn.decompositin.pca) and projected the single cells onto the same embedding. For individual single- and pseudobulk cell, we calculated the euclidean distances to cell group centers

Adjusted Mutual information (AMI) and Completeness: For clustering comparison between single- and pseudobulk cells, we performed K-means clustering (using k=7) and compared clusters to ground truth i.e. 7 reference cell groups. The AMI (sklearn.metrics.ajusted_mutual_information_score) and Completeness (sklearn.metrics.completeness_core) is calculated on RAS of individual regulons from single- and pseduobulk cells. Similarly, the RAS correlation is quantified between single- and pseduobulk cells for global and individual cell groups.

Gini Coefficient: To measure equality of RAS in classifying individual and global cell groups, we calculate Gini coefficient of RAS per regulon between both single- and pseudobulk cells.

$$G= \frac{\sum_{i=1}^{n} \left( 2i-n-1 \right) x_{i}}{n\sum_{i=1}^{n} x_{i}}$$

**Comparison between integrated and individual mouse atlases**

For clustering comparison between integrated and individual atlas, we performed k-means clustering (using k=7) and calculate silhouette score (‘sklearn.metrics.silhouette_score’), by comparing with ground truth i.e. 7 reference cell groups.

**Regulon modules and regulon networks**

Connection specificity modules and network: The Connection Specificity Index (CSI) is calculated for each pair of regulon (from Pearson correlation coefficient) and is a measure to identify regulatory partners [[12,13]](https://paperpile.com/c/JqQ5C9/sNH1L+SZHnU).

The CSI for two nodes A and B is calculated by:

$${CSI}_{AB}=1-\frac{\#nodes connected to A or B with PCC\geq{PCC}_{AB}-0.05}{n_{y}}$$

Where the Pearson correlation coefficient (PCC) is the interactional correlation between A and B.

To identify regulon modules (Fig 1F), we use hierarchical clustering (scipy.cluster.hierarchy.fcluster) on regulon linkage matrix (scipy.cluster.hierarchy.linkage) using method ‘average’ to calculate Euclidean distances between clusters. The Pairwise distance between regulons is calculated by ‘scipy.spatial.distance.pdist’ with Euclidean metric. We filter select regulon modules i.e. co-active regulons (regulons pairs) with connection specificity index (CSI) greater than 0.7 and project on a force directed graph, coloured by regulon modules (Fig 1G).

**Functional analysis of regulon modules**

Gene Ontology (GO): We use ClusterProfiler (v3.10.1) for GO analysis (Biological Processes) [[14]](https://paperpile.com/c/JqQ5C9/IEkhF). For significant GO terms within the regulon module, we use enrichGO function considering all unique genes within the regulon module as gene set. The GO comparison across modules was done using compareCluster function. Significant terms are selected using p-value cut-off (p<0.05) after adjusting for multiple testing using Benjamini-Hochberg correction.

Pathway Analysis: The regulons within individual modules are directly used for pathway analysis using Reactome (<https://reactome.org/PathwayBrowser/>) with default parameters [[15]](https://paperpile.com/c/JqQ5C9/hl6px). For each module, we quantify the significantly enriched terms (p-value<0.05) within each high-level pathway term (for example: Immune System, Metabolism, Developmental Biology etc.,). The terms and relationships were downloaded from Reactome directly (Pathways:<https://reactome.org/download/current/ReactomePathways.txt> and Relationships:<https://reactome.org/download/current/ReactomePathwaysRelation.txt>).

**Batch effect correction**

We applied two different batch correction methods on TM-10x and TM-SS2 atlases. We used pseduobulk cells from the spleen, which was profiled by both atlases.

MNN-correct: We corrected the expression space of the two atlases using the Scanpy implementation of MNN-correct (‘scanpy.pp.mnn_correct’) with parameters (svd_dim = 5 and k = 10), using the two atlases as batch key.

BBKNN: We corrected the neighbourhood graph of the two altases also using the Scanpy implementation (‘bbknn’ library version 1.3.1, with parameters (neighbors_within_batch=10, n_pcs=10, trim=50), using the two atlases as batch key.

Non-corrected: The non-corrected expression space of the two atlases was created by concatenating the individual Scanpy AnnData objects (‘scanpy.AnnData.concatenate’ with join=’inner’)

For both the non-corrected and batch corrected data, we compute regulons using pySCENIC CLI that includes RCisTarget (database: ‘mm9-tss-centered-10kb-7species’) for cross matching and regulon pruning. For regulons identified in both non-corrected and batch corrected data, we compute the spearman correlation of RAS between datasets. For each regulon predicted in the batch correction datasets, we compute Jaccard index (sklearn.metrics.jaccard_similarity_score) as a measure of composition similarity to the non-corrected dataset.

$$J\left( A, B \right)=\frac{\left| A \cap B \right|}{\left| A \cup B \right|}=\frac{\left| A \cap B \right|}{\left| A \right|+ \left| B \right|-\left| A\cap B \right|}$$

**Network validation**

STRING: The experimental annotated and scored Protein-Protein interactions (PPi) were downloaded from the static STRING database (<https://stringdb-static.org/download/protein.links.v11.0.txt.gz>) alongside their ‘Combined score’, which is a measure of confidence of interaction. We classified the PPi and CSI in 20 and 10 percentile bins respectively, based on ‘Combined score’, and compared the regulon network node-edges pairs.

OGEE: The OGEE gene essentiality table was retrieved from <http://ogee.medgenius.info/file_download/gene_essentiality.txt.gz>, and gene identifier to name mapping was performed using <http://ogee.medgenius.info/file_download/genes.txt.gz>. We only considered mouse genes for analysis. The association significance between Gene essentiality and integrated regulon network are calculated using Fisher's exact test against the entire OGEE database.

**Regulon importance and integrated network features**

Having constructed the integrated regulon network, the network features (‘Degree’, ‘Closeness centrality’ and ‘Eigen centrality’) are calculated using Gephi (0.9.2) with default parameters.

**Comparison of regulon motifs**

For each individual regulon across the integrated network, we obtained the TF binding motif from JASPAR [[16]](https://paperpile.com/c/JqQ5C9/fXkO1). We utilised a published database containing Pearson correlations between TFs position weight matrix (PWM) [[17,18]](https://paperpile.com/c/JqQ5C9/IS1ES+QDdxu) and subset, visualised the TFs from integrated regulon network.

**Comparison with alternative GRN (regulon) scoring method**

We used VIPER (version 1.16) [[19]](https://paperpile.com/c/JqQ5C9/qpBe), as an alternative to scoring regulons with AUCell. To facilitate comparison, we used the regulons inferred by GRNBoost and RCisTarget on B-cells from the TM-10X cell atlas (222 regulons, 10119 targets and 78742 interactions), applied

VIPER (‘rowTtest’) to get B-cell signatures and compared to other cell types (z-score). The t-test null model was made with 20 permutations and reposition, and the normalized enrichment scores (NES) are computed using ‘msviper’ function. We compare VIPER NES with regulon specificity score (RSS) as described in [12].

**Comparison with alternative GRN inference method**

We compared our atlas-scale GRN inference using SCENIC with an alternative published GRN method ‘bigSCale’ [[20]](https://paperpile.com/c/JqQ5C9/LSHXH) on TM-10X atlas considering the same gene-set of 11245 genes. We used the most recent bigSCale version 2 ‘compute.network’ with parameters (speed.preset=’fast’ and clustering=’direct’) and default Pearson correlation cut-off (R=0.9) for network construction. bigSCale2 captures 117 out of 174 regulons from our SCENIC consensus network. Using Jaccard index (sklearn.metrics.jaccard_similarity_score), we also quantify the regulon composition overlap between bigSCale2 and our SCENIC consensus network.

**Computational infrastructure**

The computational analysis was performed on DeIC National High-Performance Computing (HPC) cluster (ABACUS 2.0) with each node consisting of two Intel E5-2680v3 CPUs with each 12 cores and with 64 or 512 GB RAM. Dask was used to parallelize compute intensive processes across several nodes [[21]](https://paperpile.com/c/JqQ5C9/LLLE8).

**Supplementary References**

1. [Tabula Muris Consortium, Overall coordination, Logistical coordination, Organ collection and processing, Library preparation and sequencing, Computational data analysis, Cell type annotation, Writing group, Supplemental text writing group, Principal investigators (2018) Single-cell transcriptomics of 20 mouse organs creates a Tabula Muris. *Nature* **562**: 367–372.](http://paperpile.com/b/JqQ5C9/7gzKn)

2. [Han X, Wang R, Zhou Y, Fei L, Sun H, Lai S, Saadatpour A, Zhou Z, Chen H, Ye F, et al. (2018) Mapping the Mouse Cell Atlas by Microwell-Seq. *Cell* **173**: 1307.](http://paperpile.com/b/JqQ5C9/Fnvuu)

3. [Olsson A, Venkatasubramanian M, Chaudhri VK, Aronow BJ, Salomonis N, Singh H, Grimes HL (2016) Single-cell analysis of mixed-lineage states leading to a binary cell fate choice. *Nature* **537**: 698–702.](http://paperpile.com/b/JqQ5C9/kiw2W)

4. [Chen H, Albergante L, Hsu JY, Lareau CA, Lo Bosco G, Guan J, Zhou S, Gorban AN, Bauer DE, Aryee MJ, et al. (2019) Single-cell trajectories reconstruction, exploration and mapping of omics data with STREAM. *Nat Commun* **10**: 1903.](http://paperpile.com/b/JqQ5C9/LKUvC)

5. [Wolf FA, Angerer P, Theis FJ (2018) SCANPY: large-scale single-cell gene expression data analysis. *Genome Biol* **19**: 15.](http://paperpile.com/b/JqQ5C9/KYRMm)

6. [Kowalczyk MS, Tirosh I, Heckl D, Rao TN, Dixit A, Haas BJ, Schneider RK, Wagers AJ, Ebert BL, Regev A (2015) Single-cell RNA-seq reveals changes in cell cycle and differentiation programs upon aging of hematopoietic stem cells. *Genome Res* **25**: 1860–1872.](http://paperpile.com/b/JqQ5C9/tX2PW)

7. [Kiselev VY, Yiu A, Hemberg M (2018) scmap: projection of single-cell RNA-seq data across data sets. *Nat Methods* **15**: 359–362.](http://paperpile.com/b/JqQ5C9/2y9jE)

8. [Aibar S, González-Blas CB, Moerman T, Huynh-Thu VA, Imrichova H, Hulselmans G, Rambow F, Marine J-C, Geurts P, Aerts J, et al. (2017) SCENIC: single-cell regulatory network inference and clustering. *Nat Methods* **14**: 1083–1086.](http://paperpile.com/b/JqQ5C9/Y5vWg)

9. [Becht E, McInnes L, Healy J, Dutertre C-A, Kwok IWH, Ng LG, Ginhoux F, Newell EW (2018) Dimensionality reduction for visualizing single-cell data using UMAP. *Nat Biotechnol*.](http://paperpile.com/b/JqQ5C9/XBPrp)

10. [Mathieu Bastian and Sebastien Heymann and Mathieu Jacomy (2009) Gephi: An Open Source Software for Exploring and Manipulating Networks. In.](http://paperpile.com/b/JqQ5C9/nTAYb)

11. [Jacomy M, Venturini T, Heymann S, Bastian M (2014) ForceAtlas2, a continuous graph layout algorithm for handy network visualization designed for the Gephi software. *PLoS One* **9**: e98679.](http://paperpile.com/b/JqQ5C9/BHWyR)

12. [Suo S, Zhu Q, Saadatpour A, Fei L, Guo G, Yuan G-C (2018) Revealing the Critical Regulators of Cell Identity in the Mouse Cell Atlas. *Cell Rep* **25**: 1436–1445.e3.](http://paperpile.com/b/JqQ5C9/sNH1L)

13. [Fuxman Bass JI, Diallo A, Nelson J, Soto JM, Myers CL, Walhout AJM (2013) Using networks to measure similarity between genes: association index selection. *Nat Methods* **10**: 1169–1176.](http://paperpile.com/b/JqQ5C9/SZHnU)

14. [Yu G, Wang L-G, Han Y, He Q-Y (2012) clusterProfiler: an R Package for Comparing Biological Themes Among Gene Clusters. *OMICS: A Journal of Integrative Biology* **16**: 284–287.](http://paperpile.com/b/JqQ5C9/IEkhF)

15. [Jassal B, Matthews L, Viteri G, Gong C, Lorente P, Fabregat A, Sidiropoulos K, Cook J, Gillespie M, Haw R, et al. (2020) The reactome pathway knowledgebase. *Nucleic Acids Res* **48**: D498–D503.](http://paperpile.com/b/JqQ5C9/hl6px)

16. [Fornes O, Castro-Mondragon JA, Khan A, van der Lee R, Zhang X, Richmond PA, Modi BP, Correard S, Gheorghe M, Baranašić D, et al. (2020) JASPAR 2020: update of the open-access database of transcription factor binding profiles. *Nucleic Acids Res* **48**: D87–D92.](http://paperpile.com/b/JqQ5C9/fXkO1)

17. [Præstholm SM, Siersbæk MS, Nielsen R, Zhu X, Hollenberg AN, Cheng S-Y, Grøntved L (2020) Multiple mechanisms regulate H3 acetylation of enhancers in response to thyroid hormone. *PLoS Genet* **16**: e1008770.](http://paperpile.com/b/JqQ5C9/IS1ES)

18. [Madsen JGS, Rauch A, Van Hauwaert EL, Schmidt SF, Winnefeld M, Mandrup S (2018) Integrated analysis of motif activity and gene expression changes of transcription factors. *Genome Res* **28**: 243–255.](http://paperpile.com/b/JqQ5C9/QDdxu)

19. [Alvarez MJ, Shen Y, Giorgi FM, Lachmann A, Belinda Ding B, Hilda Ye B, Califano A (2016) Functional characterization of somatic mutations in cancer using network-based inference of protein activity. *Nature Genetics* **48**: 838–847.](http://paperpile.com/b/JqQ5C9/qpBe)

20. [Iacono G, Massoni-Badosa R, Heyn H (2019) Single-cell transcriptomics unveils gene regulatory network plasticity. *Genome Biol* **20**: 110.](http://paperpile.com/b/JqQ5C9/LSHXH)

21. [Rocklin M (2015) Dask: Parallel Computation with Blocked algorithms and Task Scheduling. *Proceedings of the 14th Python in Science Conference*.](http://paperpile.com/b/JqQ5C9/LLLE8)
